# Risk of contrast induced nephropathy in the critically ill: a prospective, case matched study

**DOI:** 10.1186/cc11317

**Published:** 2012-04-25

**Authors:** Cynthia M Cely, Roland MH Schein, Andrew A Quartin

**Affiliations:** 1 Division of Pulmonary, Critical Care, and Sleep Medicine, University of Miami Miller School of Medicine, 1600 NW 10th Ave, Miami, FL, USA 33136; 2 Miami Department of Veterans Affairs Medical Center, 1201 NW 16th St, Miami, Florida, USA 33125

## Abstract

**Introduction:**

Computerized tomography is frequently employed in the critically ill, often using intravenous radiocontrast material. Many of these patients have clinical features that are considered risk factors for contrast induced nephropathy, but are simultaneously at risk for renal injury from other factors related to their acute illnesses. The attributable risk for renal dysfunction from radiocontrast exposure has not been well quantified in this population.

**Methods:**

A prospective matched cohort study was conducted of patients scanned with or without radiocontrast enhancement while receiving intensive care in a Veterans Affairs Medical Center. Patients were matched for pre-scan measured creatinine clearance, diabetes, mechanical ventilation, and vasopressor use. Measured clearance was followed for three days after scanning. Evolution of nephropathy, as determined by change in measured clearance, was compared within matched pairs.

**Results:**

Fifty-three pairs of patients satisfied matching criteria. Unmatched characteristics were similar among the pairs, including serum creatinine variability during the week preceding scanning (67 ± 85% among contrast recipients, 63 ± 62% among others) and clinical risk factors for renal failure. In 29 pairs, pre-scan measured clearances were less than 60 mL/minute/1.73 m^2^. Following scanning, measured clearance declined by at least 33% in 14 contrast and 19 non-contrast patients (95% confidence interval for contrast associated difference in nephropathy rates -27% to 9%), while a 50% reduction in clearance persisted three days after scanning in three contrast and nine non-contrast patients (95% confidence interval for difference in rates -25% to 2%).

**Conclusions:**

Among established intensive care unit patients declines in glomerular filtration following contrast-enhanced scanning are common, but these changes are far more likely to be attributable to factors other than the contrast exposure itself. The upper bound for the incidence of contrast induced renal injury lasting even three days was 2% in the population studied.

## Introduction

Computerized tomography (CT) is invaluable for the management of critically ill patients. While the intravenous administration of iodinated radiographic contrast media (RCM) may be helpful or essential for adequate imaging, the potential complication of contrast induced nephropathy (CIN) is a significant concern. While it has no universally accepted definition, CIN typically refers to an at least modest decline in the glomerular filtration rate (GFR) occurring in the first days following RCM exposure. Its pathophysiology remains uncertain, with proposed mechanisms including medullary hypoxia, free radical generation, and direct tubular toxicity [1].

Depending upon the population studied, CIN incidence has ranged from 1% to more than 30% [2-12]. Risk factors include impaired renal function, diabetes, anemia, and hypotension [13-17], all common among ICU patients. Critically ill patients have therefore been presumed to be at relatively high risk for CIN [18-22]. However, analyses of CIN risk factors have focused predominantly on patients undergoing cardiac catheterization and have generally not included unexposed comparator populations. The applicability of these findings to critically ill patients undergoing CT scanning is therefore unknown.

The presumption that critically ill patients are at high risk for CIN has clinical consequences. Perhaps most importantly, it may influence how often physicians avoid exposing their critically ill patients to RCM during CT scanning even when it would provide better imaging. Furthermore, when contrast enhancement is utilized a variety of measures may be employed in an attempt to avert CIN, occasionally with unwanted consequences of their own. It is axiomatic that development of rational strategies to prevent CIN, either by prophylaxis or avoidance of RCM exposure altogether, requires accurate estimates of CIN incidence in populations of interest.

CIN is usually recognized by a rise in serum creatinine, with many investigators using as a threshold a 44 μmol/L (0.5 mg/dL) absolute increase or a 25% relative increase from before exposure. Identification of CIN among ICU patients using serum creatinine is problematic for several reasons: volatility of filtration with dynamic clinical states; rapidly changing volumes of distribution, from both disease and therapy; and varying creatinine production. These may in part explain disparate reports of CIN incidence among ICU patients [6,23,24]. The measured creatinine clearance (mCr_Cl_), a routinely available test used in trials of critically ill patients [25], intrinsically compensates for these phenomena and may provide better estimates of GFR in the ICU setting [26-28].

As the initial phase of a planned program to investigate the possible utility of very early markers of CIN in the critically ill, we undertook a prospective study of ICU patients requiring CT scanning to better quantify their risk of developing the syndrome and, in particular, to determine whether it could be identified reliably in individual patients. We opted to perform serial measurements of mCr_Cl_, before and after scanning, to identify changes in renal function with high accuracy. Because lability of renal function may lead to over-diagnosis of CIN even among less acutely ill patients [29-31], we used matched ICU patients scanned without contrast enhancement as comparators.

## Materials and methods

Subjects were recruited from the medical and surgical ICUs of the Miami Department of Veterans Affairs Medical Center from 12 October 2004 through 2 December 2006. The Human Studies Subcommittee of the Research and Development Committee (the Institutional Review Board of the medical center) approved the study, permitting urine collections before informed consent, but with consent required for sample analysis and inclusion in the study.

CTs were considered evaluable if done while the patient was in the ICU, a urine collection on that day was completed before the scan and the patient was expected to remain in the ICU with an indwelling urinary catheter for the succeeding three days.

### Urine samples for creatinine clearance

Urine was collected nightly from all ICU patients with indwelling urinary catheters who were not receiving renal replacement therapy, with a standing order to start collections at approximately 11:00 PM. Exact start times were recorded on data sheets supplied for the study. Collections were ended the next morning, with the exact completion time also recorded. Urine collection intervals thus included the time of routine serum creatinine sampling for ICUs in our institution. Collected urine volumes were measured with laboratory grade graduated cylinders.

For the few patients transferred out of the ICU within three days of a study CT, urine collections were continued in the same fashion on the ward.

### Data collection

Age, sex, ethnicity, height, weight and history of hypertension, diabetes, liver disease and congestive heart failure were recorded. The last serum albumin and hemoglobin measurements before scanning were documented, along with highest and lowest serum creatinine values from the preceding week. All clinical measurements of serum creatinine and urea nitrogen, along with the times samples were obtained, from 48 hours before to 72 hours after scanning, were logged. Data on exposure to nephrotoxins and drugs that block tubular creatinine secretion (cimetidine and trimethoprim) and administration of agents proposed as prophylaxis against CIN, including sodium bicarbonate and N-acetylcysteine, were collected.

The body area scanned, along with the type and volume of RCM, was noted. Urine output from the three hours and calendar day before scanning, and net fluid balance from the 12 hours and calendar day before scanning, were recorded. Urine outputs and fluid balances from the scan day and the succeeding three days were also recorded. Hemodynamic data included lowest blood pressure during the 24 hours before scanning, hours from last vasopressor use to the time of the scan, and lowest blood pressures and hours of vasopressor use from the three consecutive 24 hour periods following scanning.

### Study outcomes

The principal endpoint, chosen to be very sensitive to even modest effects of RCM exposure, was a 33% reduction in mCr_Cl _relative to day 0 on any of the three subsequent days. mCr_Cl _was calculated using linear interpolation of serum creatinine measurements to estimate serum creatinine at the midpoint of urine collection periods and was considered 0 mL/minute in the event of death.

Secondary endpoints included a 50% reduction in mCr_Cl _at any time, a 33% reduction in mCr_Cl _at three days after scanning and, most relevant clinically, a 50% reduction in mCr_Cl _persisting three days after scanning. Deterioration according to the Acute Kidney Injury (AKI) Network classification of renal disease [32], formulated after this investigation was initiated, was later added as an endpoint. (AKI Network criteria for oliguria were only considered satisfied if present when averaged over full days, rather than the six or 12 hour periods permitted by the Network guidelines, because of the pre-existing data format.) Relative changes in Cockcroft-Gault (CG) [33] and six variable Modified Diet in Renal Disease (MDRD) [34] estimated GFR were also determined.

### Analysis

Patients who underwent a CT with RCM were paired with a patient scanned without RCM if the following could be matched: day 0 mCr_Cl_, adjusted for body surface area, within 10%; vasopressor dependency within four hours preceding the scan; requirement for invasive ventilatory support at the time of scanning; and history of diabetes mellitus. Additional restrictions imposed by the computerized matching algorithm included: scans were only eligible if patients did not have other RCM exposures from seven days before until three days after the scan; and patients could contribute only one matched scan.

Differences in dichotomous variables between matched RCM and non-RCM patients were evaluated using McNemar's test with the binomial distribution, with confidence intervals (CIs) calculated using Newcombe's modification of the Wilson score interval [35]. The significance of differences in continuous variables was evaluated using the paired t test or Wilcoxon's test, depending upon the distribution of differences within pairs. Analyses were done using NCSS 2004 (Kaysville, UT, USA) and PASW Statistics 17.0 (SPSS, Chicago, IL, USA).

## Results

### Pre-scan data

**Figure 1:**
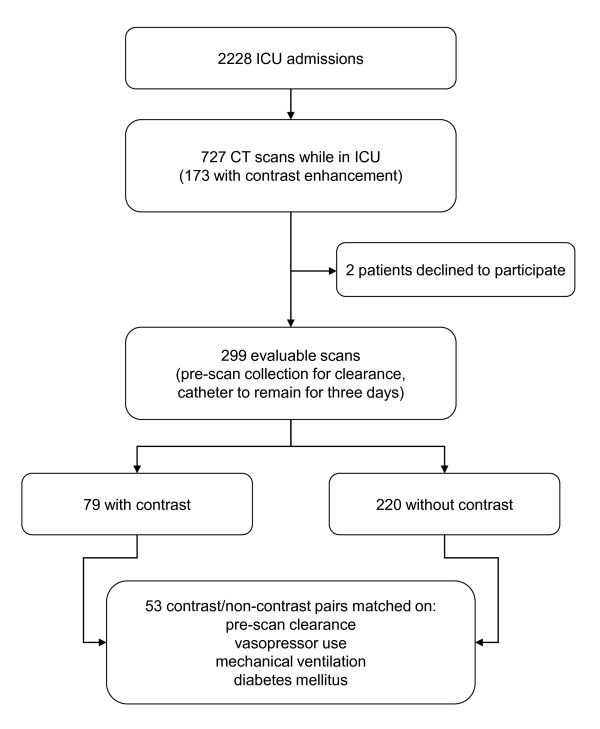
**Screening, enrollment, and matching schema**.

Throughout the study period 727 CT scans, 173 using RCM, were performed during 2,228 ICU admissions. Two patients with otherwise evaluable scans declined to participate, leaving 185 patients with 299 evaluable scans. A total of 73 patients received RCM for 79 evaluable scans, and matches were found for 53 of these patients (Figure 1). Matched and unmatched pre-scan profiles are provided in Table 1. Among matching criteria, invasive ventilation was employed for 22 (42%) pairs, and vasopressors within the four hours preceding scanning in two (4%) pairs. Fifteen (28%) pairs had diabetes mellitus. The measured creatinine clearances were 66 ± 30 mL/minute/1.73 m^2 ^(mean ± SD) and 65 ± 29 mL/minute/1.73 m^2 ^among the RCM and non-RCM patients respectively.

**Table 1:** Characteristics of patients at the time scans were performed.

	Matched Contrast Scans (*n *= 53)	Matched Noncontrast Scans (*n *= 53)	Unmatched Contrast Scans (*n *= 26)	Unmatched Noncontrast Scans (*n *= 167)
Matching criteria				
mCr_Cl_, mL/min/1.73 m^2^	66 ± 30	65 ± 29	97 ± 43	44 ± 33
Ventilated at time of scan	22 (42%)	22 (42%)	16 (62%)	98 (59%)
Pressor use in 4 hrs before CT	2 (4%)	2 (4%)	6 (23%)	19 (11%)
Diabetes	15 (28%)	15 (28%)	4 (15%)	62 (37%)
Age, years	70 ± 10	67 ± 13	61 ± 14	71 ± 12
Liver disease	17 (32%)	13 (25%)	5 (19%)	31 (19%)
CHF (current or Class III/IV)	5 (9%)	9 (17%)	3 (12%)	25 (15%)
S_Cr_, μmol/L				
Last value before scanning	92 ± 30	95 ± 41	64 ± 30	133 ± 84
Lowest in the preceding week	82 ± 28	85 ± 40	56 ± 21	108 ± 65
Highest in the preceding week	137 ± 80	134 ± 65	80 ± 31	171 ± 114
Last pre-scan BUN, mmol/L	7.1 ± 3.6	8.2 ± 4.6	4.6 ± 2.5	11.4 ± 7.1
Mean arterial pressure, mm Hg				
Before acute illness (baseline)	93 ± 14	92 ± 14	95 ± 13	95 ± 15
Lowest 24 hrs pre-scan	67 ± 16	63 ± 16	70 ± 13	65 ± 15
F_I_O_2 _at time of scan, %	42 ± 23	39 ± 16	52 ± 30	46 ± 21
New sepsis before scan	8 (15%)	8 (15%)	8 (31%)	22 (13%)
Body area scanned				
Head	19 (36%)	32 (60%)	4 (15%)	64 (38%)
Chest	35 (66%)	22 (42%)	16 (62%)	96 (58%)
Abdomen/pelvis	27 (51%)	10 (19%)	15 (58%)	44 (26%)
Contrast desirable		4 (8%)		17 (10%)

BUN, blood urea nitrogen; mCl_Cr_, measured creatinine clearance; CT, computerized tomography; CHF, congestive heart failure; F_I_O_2_, fraction of inspired oxygen; S_Cr_, serum creatinine.

Typical of Department of Veterans Affairs populations, 105 (99%) of the patients were men. RCM and non-RCM patients were similar with respect to disorders that have been associated with CIN, including liver disease and congestive heart failure (current or New York Heart Association class III or IV by history), present in 30 (28%) and 14 (13%) patients respectively (Table 2). Both groups had marked relative hypotension within the 24 hours preceding scanning (mean arterial pressure 26 ± 21 mm Hg below pre-illness baseline in RCM patients and 28 ± 21 mm Hg in non-RCM patients, *P *> 0.6), with 38 (36%) patients having mean arterial pressures less than 60 mm Hg (*P *> 0.4 for difference between groups). Eight (15%) patients in each group had been newly diagnosed with sepsis during the 48 hours preceding scanning. Fluid balances over the 12 hours preceding scanning were similar among patients who received RCM and those who did not (1,132 ± 1,550 mL versus 1,296 ± 2,159 mL, *P *= 0.50) (Table 3).

**Table 2:** Underlying traits, pre-scan risk factors for CIN and scan characteristics among the 53 patient pairs.

	Both Patients	Neither Patient	Contrast Patient Only	Noncontrast Patient Only	Contrast Associated Excess (95% CI)
Diabetes*^a^*	15 28%	38 72%	0 0%	0 0%	
Ventilated at time of CT*^a^*	22 42%	31 59%	0 0%	0 0%	
Pressor use in 4 hrs before CT*^a^*	2 4%	51 96%	0 0%	0 0%	
Liver Disease	4 8%	27 51%	13 25%	9 17%	-10% to 24%
CHF (current or Class III/IV)	2 4%	41 77%	3 6%	7 13%	-20% to 5%
Hypertension	22 42%	13 25%	9 17%	9 17%	-16% to 16%
New sepsis before scan	0 0%	37 70%	8 15%	8 15%	-15% to 15%
Nephrotoxin in 3 days pre-scan	6 11%	29 55%	10 19%	8 15%	-12% to 19%
Trimethoprim/cimetidine pre-scan	0 0%	47 89%	3 6%	3 6%	-11% to 11%
Body area scanned					
Head	10 19%	12 23%	9 17%	22 42%	-42% to -4%
Chest	14 26%	10 19%	21 40%	8 15%	5% to 42%
Abdomen/pelvis	7 13%	23 43%	20 38%	3 6%	15% to 46%

*^a^*Matching criterion. CHF, congestive heart failure; CIN, contrast induced nephropathy; CT, computerized tomography.

**Table 3:** Matched patient characteristics before scanning.

	Pairs	Contrast	Non-Contrast	Difference	Paired *P*
Age, years	53	70 ± 10	67 ± 13	3 ± 15	0.1578
Mean arterial pressure, mm Hg					
Before acute illness (baseline)	48	93 ± 14	92 ± 14	1 ± 20	0.7145
Lowest 24 hours pre-scan	53	67 ± 16	63 ± 16	3 ± 18	0.1935
Drop from baseline	48	26 ± 21	28 ± 21	-2 ± 28	0.6136
F_I_O_2 _at time of scan, %	53	42 ± 23	39 ± 16	3 ± 25	0.3068
12 hour pre-scan fluid balance, mL	48	1132 ± 1550	1296 ± 2159	-165 ± 2535	0.4984
Last pre-scan hemoglobin, g/L	53	95 ± 16	106 ± 22	-10 ± 31	0.0195
Serum albumin, g/L	53	25 ± 6	27 ± 7	-2 ± 9	0.1416
Days in ICU at time of scan	53	6.5 ± 9.7	5.6 ± 7.1	0.9 ± 11.7	0.7533
SOFA score	53	3.9 ± 2.4	4.2 ± 2.1	0.3 ± 3.1	0.5088

F_I_O_2_, fraction of inspired oxygen; SOFA, sequential organ failure assessment score.

The volume of RCM administered was 144 ± 17 mL, with 31 patients receiving iopromide (610 mOsm/L) and 22 iodixanol (290 mOsm/L). Patients receiving RCM were more likely to have scans of the abdomen/pelvis or chest and less likely of the head (*P *< 0.02 for each). CT angiography was performed during 22 scans.

**Figure 2:**
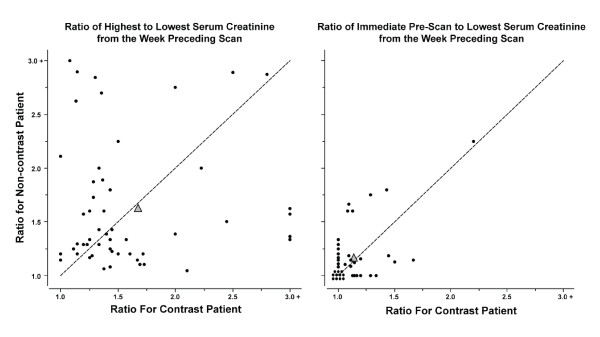
**Variability and optimization of serum creatinine before scanning**. Filled circles in the left panel represent the ratio of highest to lowest serum creatinine during the week preceding scanning in individual matched patient pairs, and in the right panel the ratio of the last serum creatinine measured before scanning to the lowest measured during the week preceding scanning. Filled triangles denote mean values for the pairs. Serum creatinine declined toward its recent minimum with pre-scanning management similarly in both groups.

Pairs were well matched with regard to pre-scan renal function (Table 4). Estimated clearances, serum creatinine and urea nitrogen, and urine flows differed little within pairs. Substantial but similar variability of serum creatinine, expressed as percent increase from the observed minimum, was seen in the week preceding scanning in both groups (67 ± 85% among RCM patients, 63 ± 62% among non-RCM patients, *P *= 0.9) (Figure 2). Serum creatinine was generally at or near its pre-scan minimum at the time of scanning.

**Table 4:** Pre-scan renal function parameters.

	Pairs	Contrast	Non-Contrast	Difference	Paired *P*
mCl_Cr_, mL/minute					
Scan day, normalized to BSA^a^	53	66 ± 30	65 ± 29	1 ± 5	0.2934
Scan day	53	78 ± 39	75 ± 34	3 ± 16	0.1768
Day before scan	24	78 ± 27	75 ± 28	4 ± 21	0.4102
Scan day calculated Cl_Cr_, mL/min					
Cockcroft-Gault formula	53	86 ± 33	90 ± 46	-5 ± 48	0.8456
MDRD formula	53	75 ± 28	76 ± 34	-1 ± 31	0.8828
S_Cr_, μmol/L					
Last value before scanning	53	92 ± 30	95 ± 41	-4 ± 37	0.4860
Lowest in the preceding week	53	82 ± 28	85 ± 40	-3 ± 37	0.6899
Highest in the preceding week	53	137 ± 80	134 ± 65	3 ± 97	0.9753
S_Cr _variability in pre-scan week, %	53	67 ± 85	63 ± 62	4 ± 106	0.9049
Last pre-scan BUN, mmol/L	53	7.1 ± 3.6	8.2 ± 4.6	-1.1 ± 5.4	0.2053
Urine vol in 3 hrs before scan, mL	53	311 ± 365	237 ± 168	74 ± 401	0.3503

*^a^*Matching criterion. BSA, body surface area; BUN, blood urea nitrogen; mCl_Cr_, measured creatinine clearance; MDRD, modification of diet in renal disease; S_Cr_, serum creatinine.

N-acetylcysteine was given to 31 (58%) RCM patients and bicarbonate to 33 (62%), with 42 (79%) patients receiving some form of putative prophylaxis. Average fluid administration exceeded three liters on the day of scanning among both contrast and non-contrast patients.

### Serial measures of renal function

**Figure 3:**
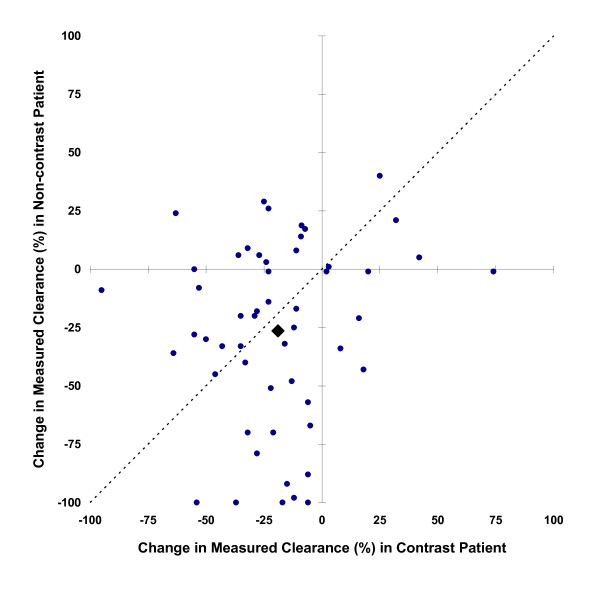
**Minimum mCr_Cl _within the three days following scanning relative to pre-scan values**. Filled circles represent individual matched patient pairs, and the filled diamond mean values for contrast and non-contrast patients. Points above the diagonal line imply greater loss of renal function in the patient who received contrast, and below the line greater loss in the patient who did not receive contrast.

**Figure 4:**
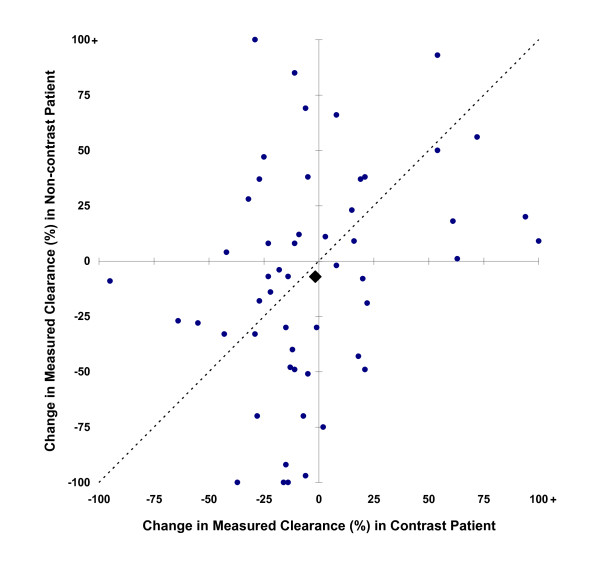
**mCr_Cl _three days after scanning, relative to pre-scan values**. Filled circles represent individual matched patient pairs, and the filled diamond mean values for contrast and non-contrast patients. Points above the diagonal line imply greater loss of renal function in the patient who received contrast, and below the line greater loss in the patient who did not receive contrast.

Clearance measurements were based on urine collections times of 9.41 ± 1.94 hours. Accompanying serum creatinine measurements were made 1.3 (IQR 1.0 to 1.5) times per study day. The principal outcome, a 33% decline in mCr_Cl _at any time during the three days following scanning, occurred in 14 (26%) RCM patients and 19 (36%) non-RCM patients (95% CI for RCM associated incidence change -27% to 9%, *P *= 0.32) (Table 5, Figure 3). mCr_Cl _at three days after scanning was less than half the pre-scan clearance in three (6%) RCM patients and nine (17%) non-RCM patients (95% CI for difference -25% to 2%, *P *= 0.08) (Figure 4). The average peak decline in mCr_Cl _was 19 ± 29% among RCM patients and 26 ± 39% among non-RCM patients (*P *= 0.23). N-acetylcysteine and bicarbonate were not associated with superior renal outcome among RCM patients (*P *= 1.0).

**Table 5:** Renal dysfunction endpoints after scanning.

	Both Patients	Neither Patient	Contrast Patient Only	Noncontrast Patient Only	Contrast Associated Excess (95% CI)
33% drop in mCr_Cl _at any time within 3 days	4 8%	24 45%	10 19%	15 28%	-27% to 9%
33% drop in mCr_Cl _persisting at day 3	1 2%	34 64%	5 9%	13 25%	-30% to 1%
50% drop in mCr_Cl _at any time within 3 days	1 2%	33 62%	7 13%	12 23%	-25% to 7%
50% drop in mCr_Cl _persisting at day 3	0 0%	41 77%	3 6%	9 17%	-25% to 2%
Required dialysis within 7 days of scan	0 0%	51 96%	1 2%	1 2%	-8% to 8%
AKI Network injury (any level) within 3 days	11 21%	20 38%	12 23%	10 19%	-14% to 21%
AKI Network stage 3 within 3 days	0 0%	37 70%	5 9%	11 21%	-26% to 4%

AKI, acute kidney injury; mCl_Cr_, measured creatinine clearance.

Estimates of GFR not utilizing urine creatinine were less sensitive than mCr_Cl_. The peak relative increase in serum creatinine within three days of scanning was 9 ± 19% among patients exposed to RCM and 16 ± 58% among those not (*P *= 0.38). Peak decline in CG estimated clearance was 6 ± 16% and 5 ± 17% (*P *= 0.26) among RCM and non-RCM patients, respectively, comparable to the values obtained using the MDRD equation (7 ± 20% and 6 ± 24%, *P *= 0.33). CG estimates identified ten patients (two who received RCM) and MDRD estimates 11 patients (four who received RCM), as having GFR fall by at least 33%.

Forty four (42%) patients satisfied AKI Network criteria for acute kidney injury, 23 of whom received RCM and 21 who did not (95% CI for difference -14% to 21%, *P *= 0.67). AKI Network level three injury, the most severe gradation, occurred in five (9%) RCM and 11 (21%) non-RCM patients (95% CI for difference -26% to 4%, *P *= 0.21).

In 29 matched pairs both patients had pre-scan mCr_Cl _less than 60 mL/minute/1.73 m^2^. Measured clearance after scanning declined by at least 33% in 5 (17%) patients who received RCM and 12 (41%) who did not (95% CI for difference -46% to 2%, *P *= 0.12). Thirty (52%) of these patients, half of whom received RCM, qualified as having acute kidney injury by AKI Network criteria. Level three injury occurred in three (10%) RCM patients and nine (31%) non-RCM patients (95% CI for difference -42% to 3%, *P *= 0.15).

The investigators judged that RCM enhancement would have been desirable for four of the matched patients (8%) scanned without it. Only one had a 33% decline in measured clearance by day three, while none of their four matched contrast-exposed patients had renal injury. Two patients received dialytic support within one week of scanning, one of whom had received RCM. Two patients, neither of whom received RCM, died within 72 hours of scanning.

Matching proved impossible for six of eight patients receiving vasopressors and RCM enhanced scanning. None of these scans were followed by a 33% decline in measured clearance.

## Discussion

We studied critically ill patients receiving RCM for CT scanning to determine their risk for CIN. Anticipating lability in renal function from critical illness itself, we compared these patients to a matched population scanned without RCM. Significant declines in renal function were frequent, but no more so among those scanned with RCM than those scanned without. It is sobering to note that absent a control population, the declines in renal function observed in the RCM exposed patients would have suggested a high CIN incidence.

To the contrary, CIN appears to have been, at most, rare in the population we studied. Our data suggest that among similar patients fewer than two per 100 should suffer a 50% loss of filtration persisting even three days as a consequence of RCM use. It is clear from our study that CIN occurs infrequently compared to other renal insults in the critically ill and loss of GFR after RCM administration, when it occurs, cannot reliably be ascribed to RCM exposure. The observed magnitude of effect is not a consequence of study size. Even a much larger study of similar patients could only be reasonably anticipated to narrow the confidence bands for significant adverse effects from RCM, but still within the upper or lower bounds of the confidence intervals we found.

Our population had many characteristics considered risk factors for CIN. Most had mCr_Cl _less than 60 mL/minute/1.73 m^2 ^before scanning. Anemia, diabetes, congestive heart failure and advanced age were common. A widely cited tool [14] for predicting CIN incidence, developed from a population undergoing cardiac catheterization, classified most of our patients as being at moderate or high risk. The predicted incidence was 17%, even without accounting for the relative hypotension and recent renal compromise prevalent among our patients. Indeed, the true incidence of nephropathy within the contrast exposed group was reasonably approximated by the model, but our control group, with similar risks but no contrast exposure, had comparable nephropathy rates.

The only randomized prospective trial of CIN prophylaxis for ICU patients, which compared theophylline with N-acetylcysteine but employed neither a control arm nor an unexposed arm [8], further underscores the need for caution when ascribing cause. In that study, a 44 μmol/L (0.5 mg/dL) rise in serum creatinine within 48 hours of imaging was defined as CIN and implicitly attributed to contrast exposure irrespective of comorbidities, and in aggregate occurred after 6% of scans. We observed a similar overall incidence of nephropathy satisfying this definition, 4%. However, all of the events in our study happened to have occurred in patients scanned without contrast (who thus had an 8% incidence), a group specifically studied because their risk profiles were comparable to patients who were scanned with contrast.

Unlike other CIN investigations, we used mCr_Cl _as the principal assessment of GFR. Both the CG and MDRD formulas presume normal creatinine generation and a static GFR. Assessments of renal function derived solely from serum creatinine suffer the same shortcomings. Among critically ill patients with potentially abnormal creatinine production [36,37] and labile filtration, mCr_Cl _may offer advantages over other commonly used estimates of GFR [38].

However, measured creatinine clearance is not without problems. It depends critically upon urine collections being accurately timed and complete. By studying patients with indwelling urinary catheters errors from incomplete voiding were minimized. Accuracy of timing was assured by recording when collections were started and ended, rather than the common practice of specifying the duration of collections in advance.

In addition to changes in GFR, creatinine based measures of renal function are sensitive to fluctuations in the tubular secretion rate of creatinine. Interval institution or cessation of drugs effecting secretion may falsely imply changes in GFR. Use of these drugs, however, was rare, and balanced between RCM and non-RCM patients. Creatinine based measures may underestimate declines in GFR if tubular secretion remains intact, particularly when GFR is initially low. While other techniques for measuring GFR, such as inulin or iothalamate clearance, avoid this shortcoming, they cannot feasibly be employed on a daily basis as this study required.

Properly obtained, mCr_Cl _provides a more accurate picture of GFR in critically ill patients than the serum creatinine or CG or MDRD formulas. However, our key finding remains unchanged irrespective of how GFR is measured, and even what level of dysfunction defines injury: the instability of renal function during critical illness overwhelms any signal from CIN, making it impossible to identify the syndrome accurately. Reinforcing this point, the pre-scanning variability of serum creatinine was of the same magnitude as that required to satisfy CIN definitions of renal injury. Such lability effectively precludes studying interventions to prevent CIN among the critically ill without at least one of the following: a biomarker more specific than GFR for CIN be identified and used as a surrogate endpoint; or very large populations are studied, without attempt to attribute individual events to contrast exposure. This caveat is also applicable to investigations of other potential etiologies of renal injury among the critically ill.

Our study has limitations. While scientifically preferable, a randomized trial of contrast exposure is impracticable, as it would mandate either suboptimal imaging or RCM exposure simply to assess toxicity, violating the principal of beneficence. We therefore employed a matched pairs design. Matching might have inadvertently paired RCM patients with unexposed patients at intrinsically higher risk for deterioration of GFR. Comparisons of other unmatched patient characteristics do not suggest this was the case. Furthermore, brain imaging, more common among non-RCM patients, tests for pathologies not generally associated with renal injury.

Because of the stringency of the matching algorithm employed, matching proved impossible for most patients receiving vasopressors. However, no unmatched RCM enhanced scan of a patient who received vasopressors was associated with even a 33% decline in measured clearance. Somehow including these patients in the analysis could only have diminished further the upper bounds for CIN incidence.

Perhaps more importantly, we could not study patients with RCM exposures shortly after ICU admission, since we required a pre-scan urine collection. These patients may well differ from those scanned later in their ICU stays. For example, there might be salutary effects of the markedly positive pre-scan fluid balances among established ICU patients. We therefore cannot exclude a possibly substantial CIN risk for patients just arriving to the ICU. We also could not study patients whose physicians felt the risk of RCM exposure outweighed the benefits. However, scanning with contrast was judged preferable from an imaging standpoint for fewer than 10% of patients scanned without contrast, including those who could not be matched.

Previously, one has had to extrapolate from other populations when judging the CIN risk faced by ICU patients and how best to manage it. Cardiac catheterization studies have provided most of these data. The critically ill patients we studied often had findings identified as CIN risk factors in cardiology populations and received RCM volumes comparable to those commonly administered during coronary angiography. However, there are potentially important differences between these populations: critically ill patients received considerably more fluid than recommended for cardiac catheterization CIN prophylaxis; exposure to certain drugs effecting kidney function, including diuretics and angiotensin pathway modulators, is likely higher in the cardiac population; and catheterization simultaneously poses a risk for cholesterol embolization. Our study indeed suggests that extending CIN findings from cardiac catheterization to CT scanning of the critically ill is unwarranted.

## Conclusions

We performed a prospective case matched study to determine the identifiability and frequency of CIN in the critically ill. Our investigation places a very low upper bound on CIN incidence in established ICU patients. The attributable risk for clinically significant renal dysfunction lasting even three days is unlikely to exceed 2%, which should inform planning for imaging studies. Furthermore, in ICU patients attribution of declining filtration to RCM exposure is unreliable because of the background prevalence of deterioration in the absence of exposure. Pending the validation of etiology specific biomarkers, this limitation will likely apply not only to investigations of CIN but also of other potential causes of acute renal injury in the critically ill as well.

## Key messages

• Among established intensive care unit patients, the risk for nephropathy lasting even three days as a consequence of radiocontrast material use appears to be small, less than 2%. This should be considered when planning imaging studies for these patients.

• Individual cases of nephropathy occurring in critically ill patients after use of intravenous radiocontrast material cannot be reliably attributed to the contrast exposure.

• Interventional studies aimed at reducing cause-specific renal injury in the critically ill will require either biomarkers more specific than glomerular filtration rate or, alternatively, very large study populations.

## Abbreviations

AKI: acute kidney injury; CG: Cockcroft-Gault; CI: confidence interval; CIN: contrast induced nephropathy; CT: computerized tomography; GFR: glomerular filtration rate; IQR: interquartile range; mCr_Cl_: measured creatinine clearance; MDRD: Modified Diet in Renal Disease; RCM: radiographic contrast material; SD: standard deviation.

## Competing interests

The authors declare that they have no competing interests.

## Authors' contributions

All authors participated in the study design, data collection, data analysis, and writing of the manuscript. All authors have read and approved the manuscript for publication.
